# Comparison of radiofrequency ablation and surgery for thyroid papillary microcarcinoma: efficacy, safety and life quality

**DOI:** 10.3389/fendo.2024.1352503

**Published:** 2024-07-12

**Authors:** Xiaoyan Kong, Liangchen Wang, Yuchen Sun, Di Zhu, Caizhe Yang

**Affiliations:** ^1^ Department of Endocrinology, Air Force Medical Center, Beijing, China; ^2^ Department of Graduate School, China Medical University, Shenyang, China

**Keywords:** papillary thyroid microcarcinoma, radiofrequency ablation, surgery, meta-analysis, life quality

## Abstract

**Objective:**

To compare the efficacy, safety and patients’ quality of life of radiofrequency ablation (RFA) and surgery in the treatment of papillary thyroid microcarcinoma (PTMC).

**Methods:**

MEDLINE, EMBASE, Cochrane, CNKI and other databases were searched for studies on radiofrequency ablation versus traditional surgery for PTMC up to October 2022. RevMan5.4 software was used for Meta-analysis.

**Results:**

10 articles were selected from 392 articles, including 873 cases of radiofrequency ablation and 781 cases of open surgery. After meta-analysis, the incidence of postoperative complications in the radiofrequency ablation group was lower than that in the surgery group, and the difference was statistically significant [OR=0.24, 95%CI (0.14,0.41), P<0.001]. There were no significant differences in lymph node metastasis rate, local recurrence rate, and new tumor rate between the two groups [OR=1.6, 95%CI (0.21, 12.41), P>0.05; OR=0.85, 95%CI (0.05, 13.8), P>0.05; OR=0.12, 95%CI (0.01, 0.98), P>0.05]. The treatment time and hospital stay in the radiofrequency ablation group were shorter than those in the open surgery group [MD=-49.99, 95%CI (-62.02, -37.97), P<0.001; MD=-5.21, 95%CI(-7.19,-3.23),P<0.001], and the cost was significantly lower than that of the traditional surgery group [SMD=-14.97, 95%CI (-19.14, -10.81), P<0.001]. The quality of life of patients in the radiofrequency ablation group was higher than that in the surgery group [MD=-1.61, 95%CI (-2.06, -1.17), P<0.001].

**Conclusion:**

Compared with traditional open surgery, radiofrequency ablation for papillary thyroid microcarcinoma has the advantages of less trauma, fewer complications, faster recovery and higher quality of life. The indications need to be strictly controlled in the treatment.

**Systematic review registration:**

https://www.crd.york.ac.uk/PROSPERO/, identifier (CRD42022374987).

## 1 Introduction

In recent years, the incidence of thyroid cancer has increased dramatically, among which the main type is papillary thyroid microcarcinoma (PTMC) with a diameter of ≤10mm. The situation in China is similar to that abroad (1). According to the Guidelines for the Diagnosis and Treatment of Thyroid Cancer (2022 Edition) (2), surgery is the preferred treatment for thyroid cancer in China, but not all patients are candidates for surgery or choose to undergo surgery. Therefore, minimally invasive treatment of thyroid microcarcinoma has attracted much attention. In this paper, the efficacy, safety and quality of life satisfaction of radiofrequency ablation and surgery were comprehensively evaluated.

## 2 Materials and methods

### 2.1 Search strategy

The study was registered in the International Prospective Register of Systematic Reviews (PROSPERO) (registration number CRD42022374987). This study searched MEDLINE, EMBASE, Cochrane, CNKI and other databases by subject headings + free words. The English search term is Thyroid Cancer, Papillary thyroid microcarcinoma, Papillary Thyroid Carcinoma, Papillary Thyroid Cancers, Radiofrequency ablation, surgery, etc. The Chinese search terms were papillary thyroid carcinoma, thyroid microcarcinoma, radiofrequency ablation, surgery, etc. The search time was up to October 2022.

### 2.2 Inclusion and exclusion criteria

Inclusion criteria: 1) studies on low-risk PTMC patients and 2) studies comparing treatment outcomes between thermal ablation and surgery.

Exclusion criteria: 1) case report or case series included <20 patients; 2) Letters, editorials, conference abstracts, consensus statements, guidelines, and review articles; 3) articles that do not focus on specific topics.

### 2.3 Data extraction and quality assessment

Two reviewers used a standard data extraction form to extract the following data from the included studies (1): study characteristics: first author’s name, publication year, study design, sample size (2); Demographic and clinical characteristics of patients: mean age, sex distribution, tumor diameter (3); Postoperative characteristics: volume reduction rate, tumor complete disappearance rate, postoperative hospital stay, perioperative cost, complication rate, operation time, recurrence rate and Thyroid Health-related Quality of Life (THYCA-QOL) (4). Postoperative complications.

Two reviewers independently assessed the methodological quality of the included studies using the Newcastle-Ottawa Scale (NOS), and disagreements were resolved with a third reviewer. The NOS scale is suitable for evaluating case-control studies and cohort studies. It has 8 items in 3 parts to evaluate cohort studies and case-control studies. These include:

Selection:

1) Representative of exposed group (1 point)
2) Representative of the non-exposed group (1 point)
3) Identification of exposure factors (1 point)
4) Confirm the outcome indicators not to be observed at the beginning of the study (1 point)

Comparability:

1) The comparability of exposed group and non-exposed group was considered in design and statistical analysis (2 points)

Outcome:

1) Evaluation of outcome indicators (1 point)
2) Long enough follow-up time (1 point)
3) Integrity of exposed group and non-exposed group (1 point)

### 2.4 Statistical analysis

RevMan 5.4 software was used to analyze the indicators, and chi-square test was used to test the heterogeneity of the indicators included in the study. If the heterogeneity test was not statistically significant (I2<50%, P≥0.05), the fixed effect model was used. The count data were analyzed by Odds Ratio (OR) (95% confidence interval), and the measurement data were analyzed by Mean Difference (MD) (95% confidence interval). If the heterogeneity test showed significant heterogeneity (I2≥50%, P<0.05), then sensitivity analysis was used to find the source of heterogeneity and Meta-analysis was performed using the random effect model. P<0.05 was considered statistically significant.

## 3 Outcome

### 3.1 Results of literature search

**Figure 1:**
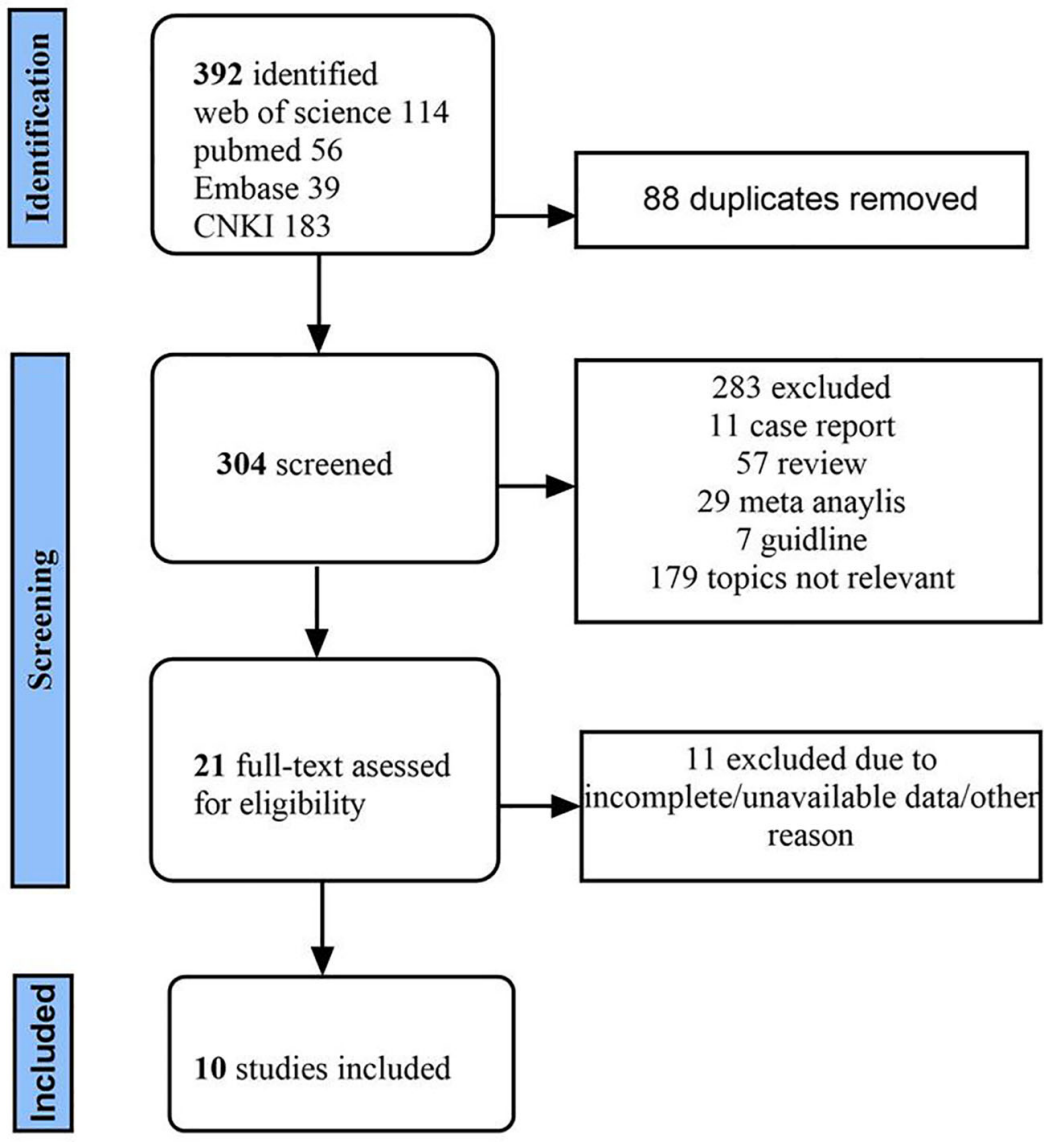
The screening flow chart.

A total of 392 literatures were retrieved from the database by means of subject headings + free words, and 88 duplicate literatures were screened out. By reading titles and abstracts, 178 unrelated studies were removed, and articles such as meta-analysis, case report and guidelines were removed. Twenty-one articles were read in full, and 10 articles were finally included according to the inclusion and exclusion criteria (3–12), The screening flow chart is shown **Figure 1**. The basic characteristics of each included article are shown in **Table 1**. According to the criteria of the NOS scale, the quality of the studies was within the acceptable range, as shown in **Table 2**.

**Table 1:** Basic characteristics of the included studies.

Included Literature	Year	Radiofrequency Ablation Group			Surgical Group		
		Number	Mean age	Sex Men/women	Number	Mean age	Sex Men/women
Gong,Hai (3)	2019	75	49.7 ± 7.4	23/52	73	48.9 ± 7.4	20/53
Lan (8)	2020	54	41.9 ± 10.2	12/42	34	42.4 ± 9.9	3/31
Zhang (5)	2020	94	45.4 ± 10.8	24/70	80	44.1 ± 9.6	20/60
Du,Kefeng (6)	2021	37	45.4 ± 6.1	13/24	37	44.6 ± 7.0	11/26
Feng,Na (7)	2021	102	47.4 ± 11.2	26/76	115	43.9 ± 10.7	23/92
Yu, Lan (8)	2021	80	42.6 ± 10.2	17/63	69	43.2 ± 10.4	5/64
Zhang,Guangxu (9)	2021	132	41.6 ± 8.9	33/99	118	43.6 ± 9.2	21/97
Zhang (9)	2021	133	45.8 ± 9.9	0/97	101	45.7 ± 10.8	0/101
Song (11)	2021	115	44.9 ± 10.4	18/97	103	45.4 ± 9.9	19/84
Zhao,Xiaoli (12)	2022	51	46.1 ± 7.4	10/41	51	47.4 ± 7.7	11/40

**Table 2:** Quality scores of the included literature.

First author,year	1	2	3	4	5	6	7	8	9	NOS
Gong,Hai,2019 (3)	1	1	1	1	0	1	1	0	0	7
Yu,2020 (4)	1	1	1	1	0	1	1	1	0	8
Zhang,2020 (5)	1	1	1	1	0	1	1	1	1	9
Du,Kefeng,2021 (6)	1	1	1	1	1	1	1	1	0	9
Feng,Na,2021 (7)	1	1	1	1	0	1	1	1	1	9
Lan,Yu,2021 (8)	1	1	1	1	1	1	1	0	0	8
Zhang,Guangxu,2021 (9)	1	1	1	1	0	1	1	1	1	9
Zhang,2021 (9)	1	1	1	1	1	0	1	1	0	8
Song,2021 (11)	1	1	1	1	0	1	1	1	1	9
Zhao,Xiaoli,2022 (12)	1	1	1	1	1	1	1	0	0	8

The surgery group included lobectomy or total thyroidectomy combined with lymph node dissection. Among them, Du Ke-feng, Feng Na and Zhao Xiao-li studied thyroid lobectomy combined with central cervical lymph node dissection (TL+CLD), and Song et al. performed total thyroidectomy combined with central cervical lymph node dissection (TT+CLD). The surgical methods of Yu Lan, Zhang2021, Zhang2020, Zhang Guangxu were determined according to ATA guidelines. The surgical group of LAN Yu et al. was divided into lobectomy group and total resection group, without mention of lymph node dissection. Gong Hai et al. did not mention the extent of surgery and the presence or absence of lymph node dissection.

### 3.2 Results of meta-analysis

#### 3.2.1 Efficacy of radiofrequency ablation and tumor progression

A total of four articles reported the efficacy of radiofrequency ablation for thyroid microcarcinoma. The efficacy was evaluated by the volume reduction rate. The patients were followed up at 1, 3, 6, 12 months and every 6 months thereafter. Feng Na (7) followed up 102 patients in the radiofrequency ablation group for 12 months, and evaluated that 100% of the nodules were completely ablated without signs of recurrence or metastasis. The effective rate of the same group was 100%, and the difference was not statistically significant. Similarly, Zhang Guangxu (9) followed up 121 of 132 patients in the ablation group, and the volume of the thyroid ablation area increased significantly on the first day and 1 month after surgery, and gradually decreased in the subsequent process. At 6 months, the ablation zone had disappeared in 37 patients (30.6%). At 12 months, the ablation zone had disappeared in 75 patients (62.0%). At the latest follow-up, the ablation area of 121 patients disappeared, and the volume reduction rate was 100%. Du Kefeng (6) et al., 37 patients in the observation group, had a preoperative tumor volume of (0. 14 ± 0. 04) mm3, the tumor volume at 1 month and 3 months after surgery was (0. 27 ± 0. 13) mm3, (0. 11 ± 0. 05) mm3. The tumor volume at 6 months after operation was (0. 07 ± 0. 04) mm3, the tumor volume at 12 months after operation was (0. 01 ± 0. 01) mm3, the tumor volume before and after operation was statistically significant (F = 74. 472, P < 0. 001). The final tumor volume reduction rate in the observation group was (93. 05 ± 3. 27) %. Zhang (10) observed the volume of ablation zone in 133 cases of ablation group at each follow-up time point, and calculated the volume reduction rate. At 18 months after the last follow-up time point, the volume reduction rate was 99.98%. A number of studies and a large number of data show that radiofrequency ablation is effective in the treatment of papillary thyroid microcarcinoma, and the tumor nodules that reach or close to the surgical group disappear completely.

**Figure 2:**
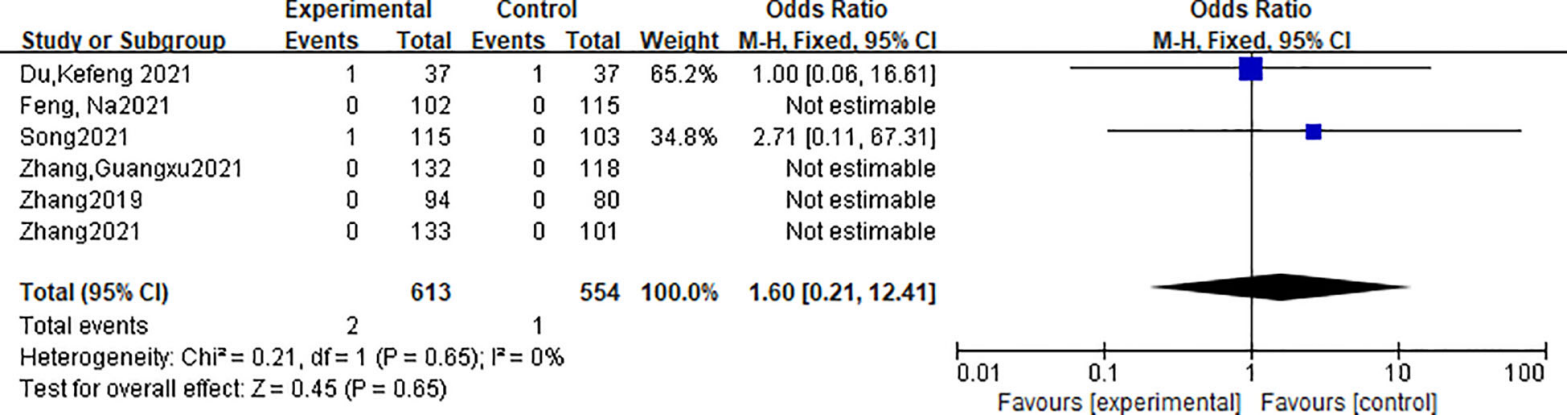
Forest plot of local recurrence rate.

A total of 6 studies reported the local recurrence rate of tumors. A total of 1167 patients were included in the literature, including 613 patients in the radiofrequency ablation group and 554 patients in the traditional thyroid surgery group. There was no heterogeneity in the indicators of the included studies (P=0.65, I2 = 0.0%), and the fixed effect model was used for analysis. Meta-analysis showed that there was no significant difference in local recurrence rate between the radiofrequency ablation group and the traditional surgery group [OR=1.6, 95%CI (0.21, 12.41), P > 0.05]. The forest plot is shown in **Figure 2**.

A total of 4 studies reported the incidence of new cancer. A total of 908 patients were included in the literature, including 461 patients in the radiofrequency ablation group and 447 patients in the traditional thyroid surgery group. There was no heterogeneity in the indicators of the included studies (P=0.91, I2 = 0.0%), and the fixed effect model was used for analysis. Meta-analysis showed that there was no significant difference in the incidence of new tumors between the thermal ablation group and the conventional surgery group. [OR=0.85, 95%CI (0.05, 13.8), P > 0.05].

**Figure 3:**
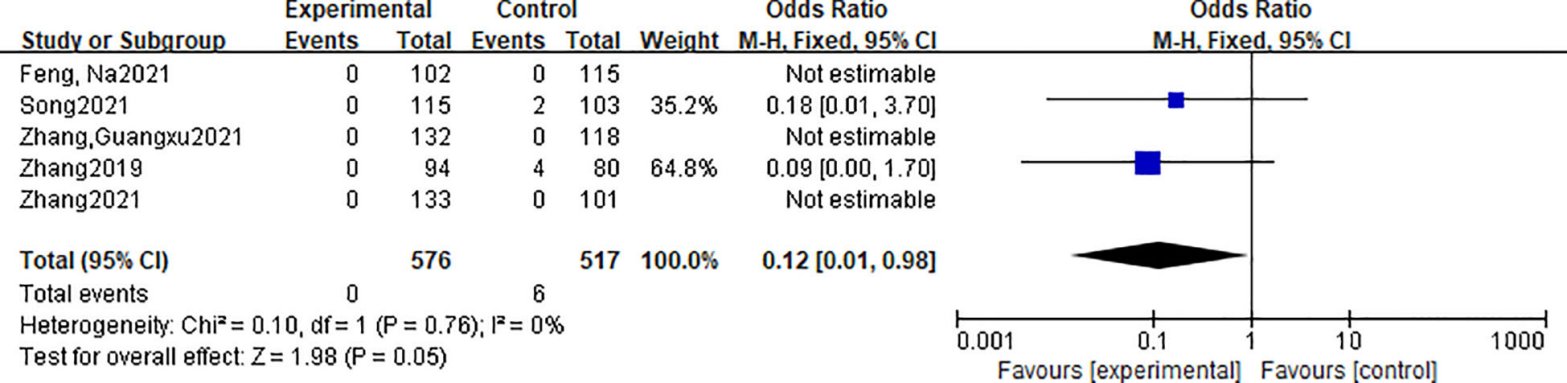
Forest plot of lymph node metastasis rate.

A total of 5 studies reported the lymph node metastasis rate. They indicated that the mode of follow-up and lymph node metastasis was 1, 3, 6, 9, and 12 months after surgery, with reexamination of thyroid ultrasound, and patients after 12 months after surgery were followed up every 6 to 12 months according to the patient’s condition. If suspicious nodules or lymph nodes were detected by ultrasound, aspiration cytology and thyroglobulin in the eluate were performed if necessary. A total of 1093 patients were included in the literature, including 576 patients in the radiofrequency ablation group and 517 patients in the traditional thyroid surgery group. There was no heterogeneity in the indicators of the included studies (P=0.05, I2 = 0.0%), and the fixed effect model was used for analysis. Meta-analysis showed that there was no significant difference in local recurrence rate between the thermal ablation group and the traditional surgery group [OR=0.12, 95%CI (0.01, 0.98), P > 0.05]. The forest plot is shown in **Figure 3**.

#### 3.2.2 Safety comparison

**Figure 4:**
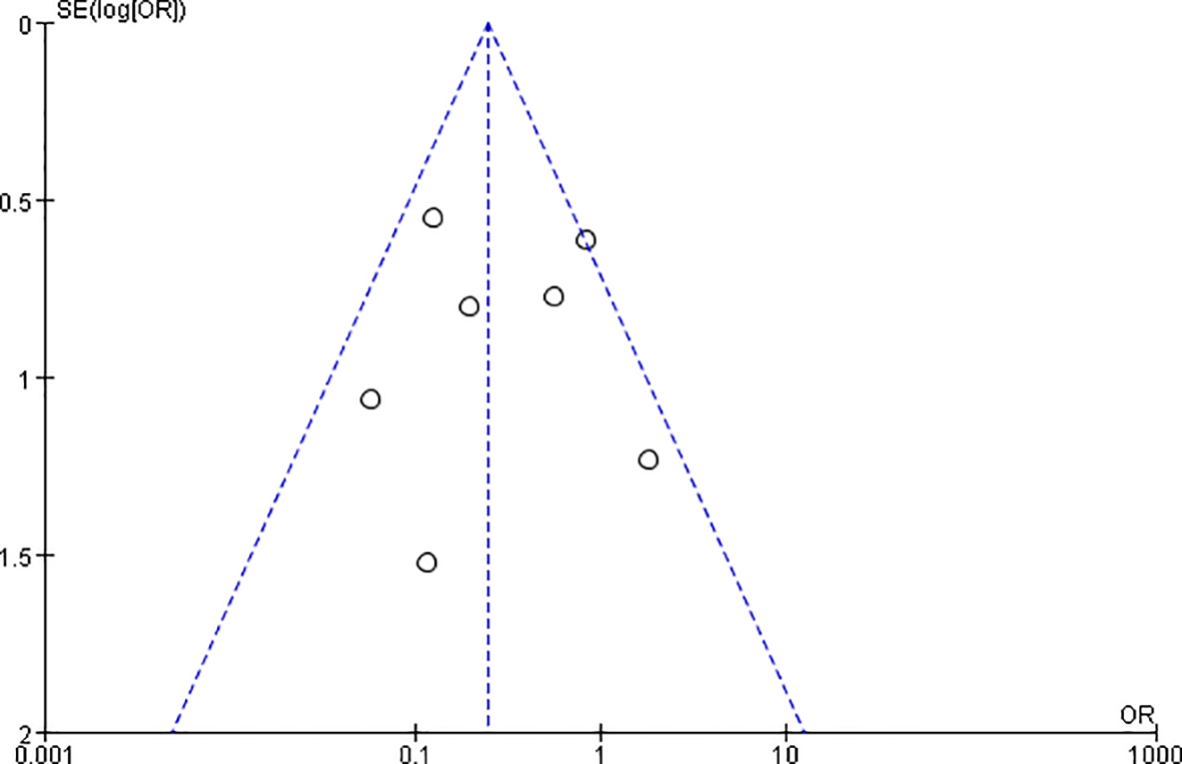
Plot of postoperative complication rates.

**Figure 5:**
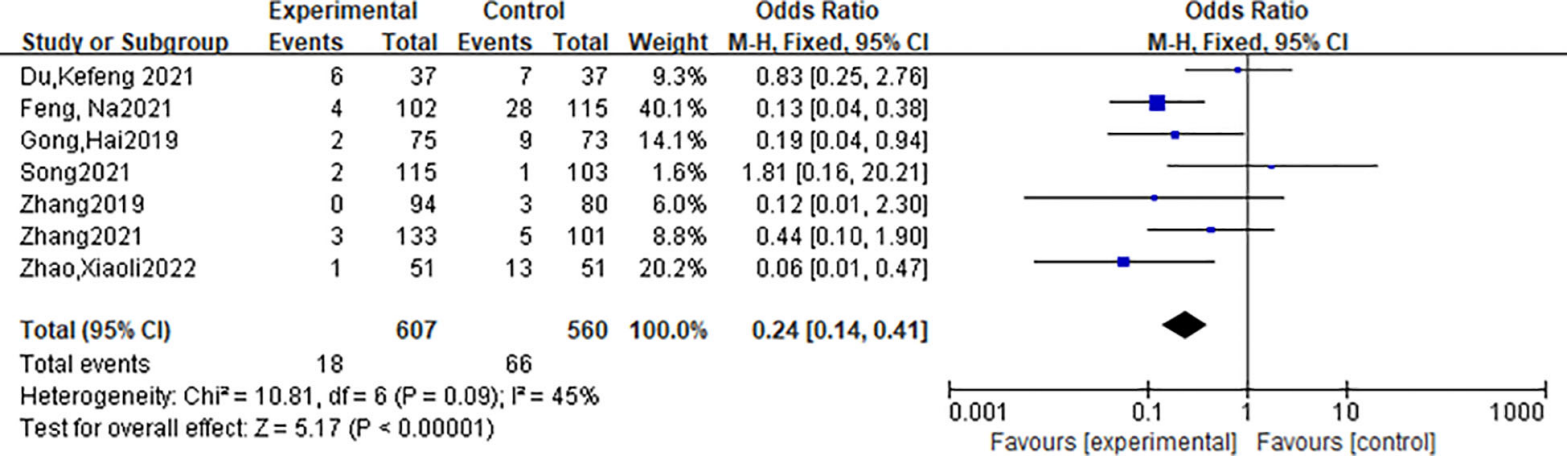
Forest plot of postoperative complication rates.

A total of 8 articles reported the postoperative complications of radiofrequency ablation and surgery group, including hoarseness, drinking cough, permanent recurrent laryngeal nerve damage, peripheral tissue damage, convulsions, incision bleeding, pain, incision infection, anesthesia vomiting, hypothyroidism, hyperthyroidism, etc. Severe complications included recurrent laryngeal nerve injury, tracheal injury, and hypoparathyroidism, while minor complications included pain, transient hoarseness, and wound infection. The impact on thyroid function was not included in the comparison, because all patients in the surgical group had permanent hypothyroidism and scar after surgery. In a study by Zhang Guangxu (9) et al., the number of patients with complications such as pain and hoarseness in the two groups was not listed. It was only reported that 1 of 132 patients in the ablation group had hypothyroidism and 3 had hyperthyroidism due to the use of Euthyroxine, while all 118 patients in the surgery group had hypothyroidism. After its removal, the heterogeneity is less than 50%, and the funnel plot is shown in **Figure 4**, with approximate symmetry on both sides indicating that the synthesized results are robust. The incidence of postoperative complications in the radiofrequency ablation group was lower than that in the surgery group, and the difference was statistically significant [OR=0.24,95%CI(0.14,0.41),P < 0.001].The forest plot is shown in **Figure 5**.

#### 3.2.3 Comparison of time and economic benefits

A total of 5 studies reported the treatment time and postoperative hospital stay. A total of 1291 patients were included in the literature, including 481 patients in the radiofrequency ablation group and 436 patients in the traditional thyroid surgery group. There was heterogeneity in the indicators of the included studies (P < 0.001, I2 > 50%), and the random effect model was used for analysis. Meta-analysis showed that the treatment time and postoperative hospital stay in the thermal ablation group were less than those in the traditional surgery group, and the difference was statistically significant [MD=-49.99, 95%CI(-62.02, -37.97),P<0.001; MD=-5.21, 95%CI(-7.19,-3.23),P<0.001]. Four studies reported the cost of treatment. There was heterogeneity in the indicators of the included studies, and the random effect model was used for analysis. Meta-analysis showed that the treatment cost of the thermal ablation group was lower than that of the traditional surgery group, and the difference was statistically significant [SMD=-14.97, 95%CI (-19.14, -10.81), P < 0.001]. The greater heterogeneity may be related to the different hospitals and geographic locations in which treatment was performed, but the results consistently showed that radiofrequency ablation was associated with shorter hospital stay, shorter treatment duration, and lower costs than surgery.

#### 3.2.4 Comparison of quality of life scores

**Figure 6:**
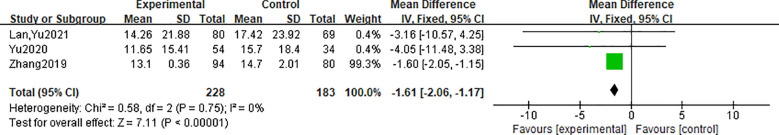
Forest plot of THYQOL score comparison.

A total of 4 studies reported the thyroid-specific quality of life score of tumors. The indicators of the included studies were heterogeneous (I2 = 99%). Sensitivity analysis excluded the literature with large heterogeneity of Song2021, and no heterogeneity was found. The THYQOL score of the RFA group was lower than that of the traditional surgery group, that is, the quality of life of the RFA group was higher than that of the surgery group, and the difference was statistically significant [MD=-1.61, 95%CI (-2.06, -1.17), P < 0.001]. The forest plot is shown in **Figure 6**.

## 4 Discussion

Due to the widespread use of ultrasound-guided fine needle aspiration cytology, the detection rate of primary papillary thyroid carcinoma (PTMC) is increasing, accounting for 50% of papillary thyroid carcinoma (13, 14). The World Health Organization (WHO) defines PTMC as thyroid microcarcinoma or less than 10 mm in diameter (1). The American Joint Committee on Cancer (AJCC) TNM system classifies T1 category (T; 1: ≤2 cm) were divided into T1a: ≤1 cm and T1b: ≤2 cm (15). There are three treatments for papillary thyroid microcarcinoma: follow-up, surgery, or minimally invasive thyroid surgery. Although several landmark studies have suggested that the prognosis of papillary thyroid microcarcinoma is good, even if the long-term survival rate of patients without intervention is not statistically different from that of patients with immediate surgery (13, 16, 17), the diagnosis of cancer may affect the quality of life of patients, and the majority of patients will choose intervention. Chinese guidelines for primary thyroid cancer (2)recommend surgery as the first-line treatment, and RFA can be applied as the preferred alternative for patients who are not suitable for surgery or refuse surgery. Although the curative effect of RFA is very good, it will lead to a variety of postoperative complications such as permanent hypothyroidism, recurrent laryngeal nerve injury, surgical scar and other shortcomings, as well as aesthetic impact. More and more studies have proved the safety and efficacy of RFA in PTMC (18–22). For example, One of the largest cohort studies to date found that Yan et al. followed 414 patients with single-focus PTMC for long-term tumor efficacy after RFA treatment after 42.15 ± 11.88 months (range, 24–69 months) (10). After RFA, 366 of 414 tumors (88.41%) completely disappeared, with a volume reduction rate of 98.81 ± 6.41%, indicating long-term efficacy in this large cohort (22).What’s more, a meta-analysis of three 5-year follow-up PTMC patients who underwent RFA involved 207 patients and 219 PTMCS, none of whom had local tumor recurrence, lymph node metastasis, or distant metastasis, or underwent delayed surgery during a mean follow-up of 67.8 months. Five new tumors developed in the remaining thyroid in four patients, four of which were successfully treated by repeated thermal ablation. The mean combined major complication rate was 1.2%, and no patient had life-threatening or delayed complications. The long-term safety of RFA has been demonstrated (20).

Therefore, ten studies were included in this study, and the radiofrequency ablation group was compared with the surgery group. In terms of efficacy, the tumor volume reduction rate of the radiofrequency ablation group was close to or reached 100% after a long enough follow-up, which was close to the efficacy of the surgery group. However, the concern that RFA treatment may affect tumor progression, such as lymph node metastasis, local recurrence, and increase in new tumors, occurred in a small number of cases among the thousands of cases in the literature, and the difference between the radiofrequency ablation group and the surgery group was not statistically significant. In terms of safety, the incidence of postoperative complications in the radiofrequency ablation group was lower than that in the surgery group, and the difference was statistically significant. The efficacy of radiofrequency ablation in the treatment of papillary thyroid microcarcinoma is similar to that of surgery, with low incidence of complications and good safety.

The study found that the treatment time and length of hospital stay in the radiofrequency ablation group were less than those in the surgery group, and the cost was lower, which was helpful to reduce the economic burden of disease. In terms of quality of life, this paper uses the thyroid specific Quality of Life scale score, which is an internationally used quality of life scale for thyroid cancer (23, 24). It was sinicized by Liu Jie et al., a Chinese expert (25) in 2019. It has been confirmed that it has good reliability and validity, and can be used as an assessment tool for the quality of life of Chinese thyroid cancer patients. It includes 24 questions summarized into seven symptom domains (neuromuscular, voice, attention, sympathetic symptoms, throat or mouth, psychological, and sensory problems) and six single items (scarring, feeling cold, tingling in hands or feet, weight gain, headache, and decreased libido). All items were scored on a 4-point scale (none, somewhat, fairly, very good). A higher score indicates that the patient complains more about the symptom. The results of meta-analysis showed that the THYQOL score of the RFA group was lower than that of the traditional surgery group, that is, the quality of life was higher than that of the surgery group, and the difference was statistically significant.

Our meta-analysis also has shortcomings, for example, there are few studies included in the quality of life score. In the future, we hope that more studies will focus on the quality of life of patients, rather than only the efficacy and safety of treatment, especially the proportion of women in patients with thyroid cancer is high, and the scar caused by surgery will affect the quality of life of patients. In addition, the surgical methods of the included studies were different, which would affect the study results. However, study heterogeneity is acceptable in the comparison of outcome measures. If there are more studies on the comparison of radiofrequency ablation and surgery in the treatment of PTMC in the future, the surgery group can be further divided into TT group and TL group for comparison.

In conclusion, RFA can fill the gap between surgery and follow-up in a minimally invasive manner. Radiofrequency ablation is the first choice for patients who are not suitable for surgery or refuse surgery. In addition, if more studies support the efficacy and safety of radiofrequency ablation, radiofrequency ablation may become the preferred treatment for patients with thyroid microcarcinoma in the future.

## Data availability statement

The original contributions presented in the study are included in the article/supplementary material. Further inquiries can be directed to the corresponding authors.

## Author contributions

XK: Writing – original draft. CY: Writing – review & editing. DZ: Writing – review & editing. LW: Writing – review & editing. YS: Data curation, Writing – original draft.
